# Knowledge and attitudes towards maternal immunization: perspectives from pregnant and non-pregnant mothers, their partners, mothers, healthcare providers, community and leaders in a selected urban setting in South Africa

**DOI:** 10.1016/j.heliyon.2021.e05926

**Published:** 2021-01-30

**Authors:** Motlatso Godongwana, Nellie Myburgh, Sunday A. Adedini, Clare Cutland, Nomasonto Radebe

**Affiliations:** a Medical Research Council: Respiratory and Meningeal Pathogens Research Unit, Faculty of Health Sciences, University of the Witwatersrand, South Africa; b Department of Science and Technology/National Research Foundation: Vaccine Preventable Diseases Unit, Faculty of Health Sciences, University of the Witwatersrand Johannesburg, South Africa; c Programme in Demography and Population Studies, University of the Witwatersrand, Schools of Public Health and Social Sciences, South Africa

**Keywords:** Maternal immunization, Vaccination, Knowledge, Attitudes

## Abstract

**Background:**

Maternal immunization has prevented millions of child deaths globally; nevertheless, incomplete vaccination remains a public health concern in South Africa, where almost half of child deaths occur during neonatal period. This study explored the knowledge and attitudes inhibiting vaccine acceptancy during pregnancy.

**Methods:**

Key informant and semi-structured interviews were conducted with pregnant women receiving antenatal care at community clinics, antenatal care staff, women enrolled in maternal immunization trials, community leaders and non-pregnant women residing in Soweto. Focus Group Discussions were also held with the mothers and husbands/partners of the pregnant women (n = 55).

**Results:**

The study established good knowledge, a positive attitude and high acceptability of maternal immunization among pregnant women, non-pregnant women, antenatal staff as well as church and community leaders. Men were the least positive about maternal immunization. Aside from antenatal staff, there was poor knowledge regarding the types of vaccinations administered and the health benefits of immunization across all the study groups. Reasons adduced for poor knowledge about the types of vaccinations include lack of communication on maternal immunization during antenatal sessions or clinic visits and power dynamics that tend to exist between healthcare workers and patients.

**Conclusion:**

Ensuring that healthcare workers provide useful information on immunization during antenatal visits as well as include men in education sessions regarding the benefit of vaccination may increase patients’ confidence and immunization uptake.

## 1 Introduction

There has been substantial progress in reducing the burden of child mortality globally. Since 1990, under-five mortality has reduced from 12.6 million to 5.3 million in 2018 (WHO, 2020a, WHO, 2020b). In 2018, the WHO European region recorded 9 deaths per 1000 live births (WHO, 2020a). However, the risk of under-five mortality remains high in Sub-Saharan Africa and low income countries. For the same year (2018), the rate of under-five mortality was 76 deaths per 1000 live births in sub-Saharan Africa (WHO, 2020a). In South Africa, under-five mortality was 33.8 deaths per 1000 live births in 2018 (UNICEF, 2020). Goal 3 of the United Nation's (UN) Sustainable Development Goals (SDGs) aims to reduce under-five mortality to 25 deaths per 1000 live births across all countries by the year 2030 all countries. To achieve this target, countries; particularly in the sub-Saharan region; need to strengthen their commitments.

Maternal immunization has been utilized for decades as a method for protection of pregnant mothers, their unborn and new born child from severe infectious diseases (Gerdts, van Drunen Littel-van den Hurk and Potter, 2016). Several vaccines are currently being recommended and used in pregnant women, including tetanus toxoid, influenza and pertussis vaccines (Böhm et al., 2019). For example, the South African National Institute for Communicable Diseases (NICD) recommends inactivated influenza vaccine to be administered to all pregnant women at any stage of pregnancy (Walaza 2014). Table 1 presents the South African immunization schedule for pregnant women.

**Table 1:** Immunization Schedule of current and future vaccines in South Africa.

Vaccine description	Schedule
Tetanus Toxoid (TT)	1^st^ pregnancy; proceeding 6 months; year 1; year 2
Influenza	Prior or during flu season
Pertussis	During the 27th through 36th week of each pregnancy
Group B Streptococcus	Under investigation
Respiratory Syncytial Virus (RSV)	Under investigation
HIV	Under investigation
Covid-19	Under investigation

In 2002, South Africa eliminated neonatal and maternal tetanus by obtaining < 1case per 1000 live births in every district (NICD 2020). Since then, the global uptake of diphtheria, tetanus and pertussis (DTP) was at 85% in 2019 (WHO 2020). Recent data shows that an estimated 84% and 77% of infants respectively received the first and third dose of diphtheria, tetanus toxoid and pertussis vaccine in South Africa.

It is estimated that about 86% of infants worldwide received three doses of diphtheria-tetanus-pertussis (DTP) in 2016 (WHO, 2017). Given the benefits accrued in tetanus immunization, low-resource countries began implementing tetanus vaccination programmes for pregnant women (Giles et al. 2018). As a result, maternal immunization, in combination with better surveillance and hygienic practices, has reduced the global tetanus mortality rate by more than 94% (Ridpath et al., 2017).

Studies conducted in the UK, US and Spain have confirmed more than 90% effectiveness of maternal pertussis vaccination in preventing pertussis infection among infants 12 weeks and younger (Baxter et al., 2017; Bellido-Blasco et al., 2017; Dabrera et al., 2015; Amirthalingam et al., 2016). In South Africa, while pertussis infection is increasingly common among infants, tetanus toxoid is the only maternal immunization that is recommended to pregnant women to prevent neonatal tetanus infection (Dangor and Lala, 2016). Evidence shows that maternal influenza vaccine is effective in preventing influenza illness in both pregnant women and infants (Fell et al., 2017). However, some countries continue to experience challenges regarding the implementation of maternal influenza vaccination programmes. Poor availability of resources and a reluctance of pregnant women to accept vaccination due to fears about adverse impact on foetal development and health have been mentioned as major barriers to the achievement of national and international targets on maternal and child health (Ortiz et al., 2012; Greenwood 2003; Munoz and Patricia 2013).

Moreover, research has shown that maternal knowledge, attitudes and beliefs play a substantial role in vaccine hesitancy (Larson Williams et al., 2018). However, research conducted in Zambia found that although mothers had poor knowledge about vaccines, they expressed positive attitude about maternal and child immunization (Larson Williams et al., 2018). For some women, traditional and religious practices inhibited the use of vaccines and/or western medicine (Larson Williams et al., 2018). A number of studies have confirmed that healthcare providers are an integral part in providing maternal information to women (Ellingson and Chamberlain 2018; Wilson et al., 2015). In the Zambian study, paternal and community rumours also had a significant influence on women's attitudes regarding immunization (Larson Williams et al., 2018). Lessons from more developed countries have shown that achieving vaccine acceptance among pregnant women and maternity healthcare professionals (HCPs) remains a considerable public health challenge (Bisset and Paterson 2018). Concerns have further risen about the paucity of research regarding the knowledge, attitudes and beliefs towards maternal immunization in low resource settings (Maher et al., 2014).

In order to achieve optimal success in current immunization programmes (administering of maternal influenza and pertussis), expand immunization programmes to include new vaccinations such as Group B streptococcus (GBS) and Respiratory Syncytial Virus (RSV) and strengthen our commitments to reduce under-five mortality to as low as 25 deaths per 1000 live deaths, it is important to obtain buy-in from all relevant stakeholders (pregnant women, maternal healthcare professionals and community members) regarding the importance of maternal immunization (Krishnaswamy et al. 2019).

In this study, we aimed at understanding knowledge, attitudes and acceptability of maternal immunization amongst pregnant and non-pregnant women, mothers and partners of these women, healthcare providers, and community members in selected urban (Soweto, Gauteng) settlements in South Africa. The use of exploratory methods is particularly important because conventional questionnaires on maternal immunization often use a "yes/no/don't know" answer format and aim to provide frequency and percentage distributions as explanations for uptake or the lack of (Awadh et al., 2014). Conventional questionnaires lack the ability to capture and explain why people think or act as they do and most questionnaires on the acceptability of immunization are often administered to women and exclude other decision influencers such as partners, parents or the larger society in which people reside (Kitano et al., 2019). Using open-ended questions to understand decision making and behaviour is important and can better assist healthcare professionals and policy makers to understand and address existing barriers to maternal immunization uptake. While the questions in SSIs are presented in a predetermined format and sequence, they allow some flexibility in the way a topic is addressed by both the interviewer and respondent. In this study, respondents were encouraged to share their thoughts and ideas rather than providing “yes” or “no” type of answers. The results in this study are part of a larger study that aims to understand the acceptability of maternal immunization in both urban and rural (Mtubattuba, KwaZulu-Natal) settlements in South Africa. These findings are important for increasing acceptancy of current and future immunization programmes essential for informing larger studies in similar and/or different contexts on acceptable entry points to introduce future immunization programmes.

## 2 Methods

### 2.1 Context

The study was conducted in Soweto (South-Western Township). Soweto is a congregation of 29 townships within the Johannesburg Metropolis in South Africa. It is inhabited by a low-income, urbanized Black-African community of Zulu, Xhosa, Pedi, Tsonga, Venda, Tswana and Sotho ethnicities (STATS-SA, 2012), mainly of Christian religious background (mostly Protestant and Charismatic). The total population is 1.4 million people, of whom 125,000 are under-5 years of age.

### 2.2 Study design and sampling strategy

We designed a qualitative exploratory study to explore the knowledge, attitudes and acceptancy of maternal immunization. Semi-structured questionnaires were developed. The qualitative data was collected through the use of semi-structured interviews (SSIs), key informant interviews (KIIs) and focus group discussions (FGDs). These methods were used in order to carefully explore the views and concerns emanating from the selected sample regarding maternal immunization uptake and acceptability. A combination of purposive and snowball sampling techniques was employed to identify potential respondents. Purposive sampling was used to select pregnant women receiving antenatal care at community clinics, women enrolled in maternal immunization trials as well as antenatal staff, church and community leaders residing in the study location. The snowballing method is useful for hard to reach population (Bonevski et al., 2014). Snowballing was used to recruit the partners of pregnant women, their mothers and some of the non-pregnant women. All the pregnant women were requested to inform their mothers and partners about the study. We recruited all partners and mothers that showed interest to participate in the study into separate focus group discussions. To obtain the sample of non-pregnant women, healthcare providers, community leaders and pregnant women were asked to inform other women that they knew about this study. SSIs were then conducted with all non-pregnant women (with and without children) that were willing and able to participate in the study. We recruited both males and females from the community of Soweto. The selection of both sexes was done to ensure that all potential influencers for women's decision making on maternal immunization are represented. However, the sample size and findings in this study are not representative of the entire Soweto population as only a subset of this community was sampled (Table 2). Nonetheless, the use of varying qualitative methods ranging from focus groups and in-depth interviews has allowed us to identify emerging themes and prevalent opinions pertaining to the knowledge, attitudes and acceptability of maternal immunization within an urban community such as Soweto. Table 2 below presents the demographic and socio-economic characteristics of the selected sample n = 55.

**Table 2:** Study population and method of data collection.

Study Participant	Sample size	Data collection method
Pregnant mothers – prim gravida & multiple	6	SSI
Non-pregnant women with/without children	10	SSI
Women enrolled in maternal immunization trials and who previously had a child with Group B streptococcus (survived or died)	10	SSI
Husbands/partners of pregnant women	1 × 6 participants	FGD
Mothers of pregnant women	1 × 6 participants	FGD
Antenatal and maternity staff from community and tertiary hospitals	7	KII
Other maternity healthcare providers such as doulas, midwives, breastfeeding consultants	5	SSI
Community leaders	5	KII

### 2.3 Data collection methods

Leveraging on our experiences in implementing Soweto Health and Demographic Surveillance System (HDSS), we secured community entry and had participants’ mobilization through our Community Advisory Board (CAB) already established in the community. The CAB assisted to book appointment with church and community leaders and other categories of participants in the community. As shown in Table 2, there were 55 study participants in total. The questionnaires used for the different categories of participants are included as a supplementary file to this paper.

The interviews were conducted by a Social Scientist and a trained Research Assistant. Each individual interview lasted around 30 min while the FGDs were approximately 60 min in duration. The study participants differed in their ethnic background, level of education, employment status and age. Table 4 presents the distribution of participants by socio-demographic characteristics. Depending on the participant's literacy level, the interviews were conducted in either English and/or in local languages. These local languages included Sepedi, isiZulu, isiXhosa and Sesotho). Interviews were recorded with the use of tape recorders. Except those conducted in English, we first translated the interviews from local language to English and then transcribed them verbatim.

### 2.4 Ethical considerations

Ethical clearance was obtained from the Human Research Ethics Committee (Non-Medical) at the University of the Witwatersrand (H18/07/03). The research objectives were explained to all study participants. Signed consent forms and verbal consent for the tape recording were obtained before commencing the interviews and FGDs. Confidentiality was maintained by not allowing any of the interviews to be accessible to anyone outside of the research team.

### 2.5 Data Analysis

All interviews were translated into English. Thematic content analysis was conducted. The transcriptions were organized under thematic headings and later developed into an ethnographic summary with illustrative quotes. Table 3 below presents some of the themes observed.

**Table 3:** Thematic and content analysis: Findings from Soweto.

Thematic Heading	Description	Content
Knowledge of maternal immunisation	Pregnant women receiving immunization. Immunization of mothers. Giving mothers injections.	‘It is to prevent diseases whilst the woman is still pregnant’ – FGD, Mothers. ‘It's all about preventing infections that can affect a baby’ – pregnant mother. ‘I think you are talking about the mother being immunized. Immunization refers to injections’ – non-pregnant woman. ‘Maternal is mother then immunisation is where by a mother like myself, she takes her child to the clinic to get their vaccines’ - Doula. ‘I think they take pills that prevent the baby from getting HIV if the mother is HIV positive. It's a pill of some sort’ – pregnant mother.
Knowledge of types of maternal immunization	Tetanus/ATT or Pertussis or Flu vaccine	We give pregnant mothers tetanus when they first book at the clinic’ – midwife. ‘The flu vaccine is given once when they come to book, but obviously if the pregnant mother has got the signs of flu’ – midwife. ‘I don't know of any’ – pregnant mother. ‘According to my experience I haven't seen one, even when my wife was still getting children there was no immunisation that was given to her on behalf of the baby’ – FGD, fathers. ‘I did not receive any vaccinations during my pregnancy. But I know of the vaccination that prevents a baby from getting HIV’ – mother, GBS positive.
Uptake & Use of maternal immunisation	Tetanus/ATT or Pertussis or Flu vaccine	‘I received some injections during my pregnancies but I do not know what they were for’ – pregnant woman. ‘I was never vaccinated. They just did the normal procedure like HIV test, that's the only thing I can remember’ – mother, GBS positive. ‘I don't remember getting any vaccination during my pregnancy. I just remember that they took some blood from me to do some tests' – pregnant woman. ‘During my pregnancy I received the flu vaccine. I don't remember receiving any other one – pregnant woman’. ‘I received only one vaccination when I was pregnant. They told me that it was for my discharge or something like that. It was an injection’ – non-pregnant woman. ‘I was attending the Chiawelo clinic, I know they vaccinated us. The first injection they gave me, they said if my blood and the babies are not same group, it shouldn't affect the baby, something like that and they injected me with that’ – non pregnant woman. ‘I have been vaccinated before but I don't know the name of the vaccine’ – pregnant woman.
Attitudes	Positive or pro immunization during pregnancy Negative or anti-immunization during pregnancy	‘Pregnant women come to the clinic and just go with the flow of what the sister is saying’ – midwife. ‘The thing is sometimes when we are given vaccines we don't ask what we are being given. You find that they just administered’ – pregnant woman. ‘‘I remember the flu vaccine. I have never heard of tetanus. Truly speaking, I also did not ask the nurses anything when I was pregnant’ – non pregnant woman.
Beliefs and misconceptions	Thoughts/perceptions about immunization Cultural perceptions Religious perceptions Myths	‘I have never come across a case where women object immunization because of religious beliefs’ –midwife. ‘I didn't take the flu vaccine because two years ago my sister received the flu vaccine from Bara. She was fine during the pregnancy but after she delivered the baby she got such a strong and bad flu – pregnant mother’. ‘Immunization is one of the ways the government uses to control people’ **–** FGD, men ‘In my Xhosa culture there are certain illnesses that they encourage us not to go to the clinic for. That instead we must use traditional medicine until I am fine – pregnant mother. ‘They need to explain to me the importance because when a person is pregnant, she doesn't just take anything. Even if you get flu or you have a headache you don't drink any medication’ – mother, GBS positive. ‘My main fear is that what if the nurses do not vaccinate us for the correct thing. How do we know that they are vaccinating us for the correct thing? That makes me fearful’ – pregnant mother.
Acceptability of maternal immunization	Intension to make use of vaccination Encouraging pregnant women to be immunized	‘With my first pregnancy I was a bit more concerned about taking things while I am pregnant. I just feared that by taking things then my child would not be okay. I worried about the side effects of taking medication and what if they affected the baby’ – pregnant mother. ‘I encourage it because if you are not taking that injection to get that immunisation it means that you putting the baby on the risk because you won't know on the day you deliver what problems you will encounter and the baby will be affected’ – Community leader. ‘I think especially since we normally say prevention in better than cure, so for preventative methods so if there could be any viruses that the baby could pick up because the mother has not been immunised- Counsellor
Factors affecting future use immunizations	Socio-economic Socio-political	‘I think the question of unemployment is a serious problem in our country’ – Counsellor. ‘Vaccination should always be free. If there was a cost, then as for the mom who goes to the clinic it would be a problem because some are unemployed or stay at home moms, some don't even have money and others are single’ – Doula ‘It comes back to cost because even if the vaccine is available and I do not have the money to get it then I will not be able to get it – pregnant mother.

## 3 Results

A total of 12 Key informant interviews (KIIs), 31 semi-structured interviews (SSI) and 2 focus group discussions (FGDs) were held among the study population. Each FGDs had six participants. A total of 45 women and 10 men were studied. Of this number, 12 participated in FGDs while 31 were SSI and 12 KII participants. The knowledge and attitudes towards maternal immunization were analysed across 5 thematic areas. This included knowledge of maternal immunisations, uptake of maternal immunization, beliefs/misconceptions, acceptability of maternal immunization and potential use of future maternal immunizations. The results are structured according to these thematic areas.

### 3.1 Socio-demographic characteristics of respondents and maternal immunization uptake

Table 4 presents the percent distribution of study participants according to their socio-demographic characteristics.

**Table 4:** Distribution of individual interview & FGD participants by select background characteristics.

Characteristics	Frequency (N)	Percent (%)
**Age**		
22–29	25	45.4
30–39	16	29.0
40 + years	14	25.4
**Gender**		
Female	49	89.0
Male	6	10.9
**Level of education**		
Some secondary	13	23.6
Completed secondary (Matric)	26	47.2
Tertiary	16	29.0
**Employment status**		
Unemployed	26	47.2
Employed	27	49.0
Self employed	2	3.6
**Race**		
Black	53	96.3
Coloured	2	3.6
**Children ever born**		
None	18	32.7
1–2	27	49.0
3+	10	18.1
**N = 55**		

The results revealed that the younger (22–29 year olds & 30–39 year olds) participants were more accepting of maternal immunization compared to the 40 + year olds. The younger participants mentioned that many of their fears regarding maternal immunization were eased because they could use search engines on the internet such as Google to obtain further information if they were uncertain. However, two of the maternity unit managers mentioned that the challenges they received were of younger pregnant mothers visiting the clinic later in their pregnancy for antenatal care and, therefore, being too late to be administered vaccinations. HCPs further reported that, in most cases, pregnant women aged 30–39 years) were more accepting of vaccinations than younger pregnant women because the younger ones rarely visited the clinics for antenatal care. In the men's FGDs, the younger participants also mentioned that they lived in an era of “responsible fathers” and, thus, encouraging their partners to immunize fit well within this role. On the other hand, while the older (40 + years old) were open to maternal immunization, most expressed that access to information regarding it was still a problem. The majority of the study participants had matric (grade 12). These participants had better knowledge regarding maternal immunization compared to those that only had some secondary education. With regards to employment status, all participants mentioned that they felt they were at a disadvantage if future maternal immunizations were to come at a cost because they would not afford to purchase them. The unemployed participants who had children and were pregnant noted that they were dependent on grant money. Even the unemployed participants expressed that they would be reluctant to pay for maternal immunization because they were used to the, already, free services provided at public clinics. Lastly, participants that had children were more open to maternal immunization compared to those that had no children. Those without children had the most fears regarding maternal immunization.

### 3.2 Knowledge and uptake of maternal immunization

We sought to understand whether participants had knowledge of maternal immunization. Overall, there was fair knowledge regarding maternal immunization particularly among women (pregnant and non-pregnant), with 4/6 pregnant women being able to explain the concept very well. The majority of the study participants broke the term down referring to “maternal” as having to do with women or mother and “immunization” being injections. All antenatal and maternity staff were able to fully explain that the term referred to vaccinations provided to pregnant women to prevent the child from infectious diseases. However, some women (2/6 pregnant; 4/10 non-pregnant) and community leaders (2/5) confused immunization with prevention of mother-to-child (PMTCT) of HIV. Knowledge of maternal immunization was poorest among partners/husbands and mothers of pregnant women. In both FGDs (mothers and partners, respectively), participants were able to partly explain the term maternal immunization. Their understanding was mainly that it was the “injections given to children”, not necessarily pregnant women. All men in the FGD had no knowledge of the types of immunizations given to pregnant women or even to the “children”.

The importance of maternal immunization was largely unknown among the study population because some of the pregnant women (3/6) and those not currently pregnant (5/10) who had at least one child reported that they were “injected” but were unaware of what the injections they received were meant for. The remaining 50% of the pregnant women said they were never given any vaccination. In the mother's focus group, only one respondent mentioned tetanus and that she remembered her daughter had received it. However, the types and importance of maternal immunization were unknown across all other study groups (men FGD and SIIs with community leaders). Our study found that only maternity or antenatal staff (7/7) were knowledgeable about the different types of maternal immunization.

All the sampled midwives in our study reported that pregnant mothers are given two vaccinations during pregnancy, namely, influenza and tetanus toxoid. However, only 1 of the 6 pregnant women interviewed and (3/10) non-pregnant women who had ever been pregnant confirmed receiving tetanus toxoid vaccine. At least 2/10 of women in maternal immunization trails and 4/10 non-pregnant reported not ever receiving the influenza vaccine. When asked if they knew their partners to have received any vaccination (tetanus or influenza), some men (2/6) reported that their wives/partners did not discuss the detail of medication they receive from the clinic with them while others (4/6) said their wives/partners had never reported receiving either of these vaccinations.

When prompted to understand reasons why uptake of maternal immunization was low, most of the women (5/6 pregnant; 7/10 non-pregnant) cited that they did not ask about immunization during antenatal visits because they trusted that their midwives were knowledgeable and experienced and would not provide them with any medication that would harm their babies. Three of seven of the antenatal staff interviewed confirmed that pregnant women attending antenatal classes/check-ups rarely asked about medications administered to them or vaccinations for that matter. Two of the antenatal unit managers stated that midwives educated pregnant women about different vaccinations during antenatal classes. However, of the pregnant women attending; or had once attended; antenatal classes at the community clinics, no mention was made about antenatal classes that included lessons on maternal immunization.

### 3.3 Beliefs and misconceptions regarding maternal immunization

After we provided an explanation of maternal immunization, the majority of study participants expressed some fear regarding immunization. The fears expressed by the studied participants were diverse. Among pregnant women; non-pregnant women who had never had a child and mothers of pregnant women, the commonly cited fear was the possibility that immunization would affect the health of the unborn child. However, most of them said that some of these fears were eased by the fact that immunizations were provided in a health facility and by professional nurses, who they believed were trained and would not in any way harm them. Some women in the FGDs went on to mention that they trusted immunizations from public health facilities as opposed to private health facilities because they have heard that some private doctors purchase their practice license and are not ‘real doctors’ but quacks that are authorised to provide vaccinations. Some men (3/6) in the FGDs had the perception that immunization was “a way used by the government to control people”. These men expressed that the prescribed injections given to children from a young were done to ensure that children grow to always obey the law and the systems of the government. One man went on to mention that he knew of a couple that have never vaccinated their child but the child grew up to be healthy. This man largely questioned the need of vaccinations in general and gained the support of a few other men in the FGDs. Given these views, these men were reluctant for their pregnant partners to be immunized. Some of the participants had a negative attitude towards the influenza vaccine. The pregnant women (3/6), non-pregnant women (4/10) and mothers of the pregnant women (1/6) reported that they knew someone who had taken the flu vaccine but still later developed cold/flue or that they had experienced this themselves. This made them question the effectiveness of vaccinations and their usefulness in general.

### 3.4 Acceptability of maternal immunization

About 80% of all study participants were in support of maternal immunization once it was explained to them. We explained that maternal immunization is the vaccination given to pregnant women to protect both the mother and the foetus from morbidity and infection. We provided examples such as tetanus toxoid, influenza vaccine and also explained that new vaccinations to prevent Group B Streptococcus (GBS) and other infections were being developed. The participants that were keenest to accept future immunization programmes were the women who had experienced their child either die or become sick as a result of GBS. These women were enrolled in a GBS trial and had found out their baby's cause of death or illness through participation in the trial. Of the 10 women enrolled in maternal immunization trials and interviewed in this study, about 40% reported having a baby that died due to GBS while another 40% had a child that was infected with GBS but survived. Women who had experienced their child infected or die as a result of GBS reported that their experience made them more cautious about taking vaccinations in their future pregnancies. The partners/husbands (2/6) of these women also expressed similar views. Community leaders, particularly the church leaders, said they encouraged women to adhere to advises and medications provided by health care professionals, including taking vaccinations were necessary. The mothers of pregnant women were accepting of maternal immunization said they continued to require reassurance from health care providers that these vaccinations were to the benefit of their children and grandchildren. Antenatal staff were also keen to hear more vaccinations are to be developed. They expressed that they required training on these diseases (GBS) and the benefits the vaccinations would provide thereof.

### 3.5 Factors affecting use of future immunizations

Most of the participants (80%) expressed that the only hindrance that may affect their use of vaccinations is if they come at a cost. They noted that all services currently provided at the public clinics were free and thus future vaccinations provided within these facilities should be made free as well. Another factor commonly raised was religion. Most study participants mentioned that some religions were against the use of vaccinations. However, when prompted to talk about their own religion, they expressed that theirs had no problem with maternal immunization. Two of the community leaders that were interviewed were pastors of local Christian churches. These participants expressed that they were in full support of maternal immunization. Only one of the six pregnant women interviewed and one of the mothers in the FGD who were Muslim mentioned that they were concerned about the ingredients used in medications and vaccinations because their religion was strict on the use of alcohol. These participants mentioned that they were cognizant about the amount of alcohol that went into medications including vaccinations and often enquired about this during consultations. However, both mentioned that the volume of alcohol included in vaccines was often limited and did not prevent them from getting immunized or immunizing their children. Within the men's FGDs, two of the participants mentioned that their culture required pregnant women to undergo and make use of specific traditional medication. However, they noted that this often does not interfere with immunization and can be done concurrently with maternal immunization. The antenatal staff said they had not experienced any challenges about patients who objected medications or vaccinations because of their cultural or religious beliefs.

## 4 Discussion

About 60% of participants in this study had some knowledge of maternal immunization. In addition, there was an overall positive attitude regarding maternal immunization among women, community and church leaders. The participants that reflected a positive attitude were either women who understood the benefit of maternal immunization to themselves, their unborn babies or children or individuals who because of their societal status as leaders, had the health interest of their constituencies at heart. Increasing acceptability of maternal immunization among the wider population requires educational interventions that address existing myths and fears and for health campaigns to market the immunization in a way that emphasises the intended benefit.

Previous studies that have established poor knowledge of maternal immunization in both low and middle income countries (Ahmed et al., 2001; Mayat et al., 2017). While the understanding of the term ‘maternal immunization’ did not occur intuitively to most of the participants in this study, the findings presented in this study show promising improvement in knowledge levels in so far as most participants being able to understand the terms “maternal” and “immunization” respectively and thereof generating an idea of the joint meaning. Studies have shown that mother's education is significantly associated with maternal immunization knowledge and uptake (Arsenault et al., 2017; Balogun et al., 2017; Chidiebere et al., 2014). We found that participants with completed secondary education (matric/grade 12) had better knowledge regarding maternal immunization compared to those with just some secondary education. However, if knowledge is assessed beyond the ability to interpret meaning, then the fairly high knowledge levels (60%) in this study remain inadequate as only few participants could provide example of maternal immunizations that they had received or knew about.

Several studies have shown that knowledge of maternal immunization is a key determinant of the uptake of maternal immunization (Eppes et al., 2013; Bushar et al., 2017). The average knowledge levels found in this study suggest a relatively low uptake of current maternal immunization programmes. The uptake of maternal immunization has been below national targets for most countries, globally. For example, the influenza vaccine uptake was 50% in the US, less than 65% in the UK and only about 2% in Hong Kong (Vojtek et al., 2018). Despite fair knowledge and a general positive attitude towards vaccinations, most pregnant women in this study had never been immunized while others, of those that have been immunized, did not know what they were vaccinated for.

Healthcare providers are a critical component in the transfer of information to patients regarding general health information including maternal immunization. However, challenges with staffing, limited resources and training stand as barriers to delivering comprehensive information during consultations. In this study, while all healthcare providers were knowledgeable of maternal immunization, its importance and the need to provide it, the findings in this study of patients not knowing the types of immunizations that are available and some reporting not ever being immunized suggest existing gaps in the current immunization programme; from both the healthcare system (affecting supply/administering of maternal immunization) and the education given to women around maternal immunization; that if not addressed, may affect the acceptability of future immunization programmes.

The success of future immunization programmes could further be hindered by several barriers including existing fears and misconceptions; misinformation and lack of adequate knowledge; religious beliefs and associated immunization costs.

The fears and misconceptions that continue to exist regarding immunization. For example, the present study found that some men believed that immunization was used to control people. The perception was that the government administered vaccinations to, especially, children from a young age in order to control their growth. This result is similar to findings from another study that found that rumours regarding immunization included that it was used as a means to control birth (Messeret et al., 2018). Research shows that ‘paternal involvement and community rumours has an influence on the attitudes of women regarding immunization (Larson Williams et al., 2018). Given that husbands/partners in this study showed poor attitude towards maternal immunization, it is important to ensure that education programmes on maternal immunization extend to include men as well. Nudging partners and well as community members to understand the benefits of maternal immunization can be a good step to ensure that they encourage their partners and women about the importance of maternal immunization. Despite that research has found that fears of receiving vaccinations during pregnancy is no longer a barrier for maternal immunization as compared to a century ago (Greenwood, 2003; Munoz and Patricia 2013), our study revealed that fears such as the possible harm of immunization on the baby continue to exist among pregnant women and their mothers. However, the perceived benefit seemed to outweigh these fears as most of these women continued to show a positive attitude.

We found that antenatal and maternity staff have an important role to play in allaying existing fears around maternal immunization by providing adequate information regarding immunization to pregnant women and all other patients attending health facilities. This is mainly because women, including mothers, trust healthcare providers to provide them with information even in situations where the patient does not ask for this information. Similar to the finding in this study, other studies have found that pregnant women trust healthcare providers to provide information on immunization (Nganga et al., 2019). Overall, women and community leaders are to the view that the medication they receive from their antenatal care providers will benefit pregnant women and their unborn children. However, it was unclear whether information on maternal immunization was included during antenatal classes or when the pregnant mothers were being vaccinated. The lack of a comprehensive introduction and explanation of maternal immunization, particularly, to pregnant mothers may prove to be problematic for current and future immunization programs because pregnant mothers may become reluctant to immunize or request to be vaccinated in the event that immunization is not offered.

There is diverse evidence on the effect of religion on immunization. Some studies that have found religion as a barrier to child immunization (Imdad et al., 2013; Pelčić et al., 2016). For example, in one of these studies, Muslim children had a greater chance of being under vaccinated (Pelčić et al., 2016). Overall we found that religious and traditional participants in this study remained intuitively positive about maternal immunization. While there were concerns expressed by Muslim participants about the amount of alcohol that goes into vaccinations, each acknowledged that the amount of alcohol used in immunizations was not a great deal to motivate or lead to non-use. In fact, one study went on to find that Muslims were more likely to support immunization than other religions (Kalok et al., 2020).

Finally, our study showed that cost was a major barrier that could affect uptake of future immunizations. The findings show an overall preference for vaccinations to be provided for free in order to ensure consistent use, especially for unemployed pregnant women or women that cannot always afford to pay for the immunization.

## 5 Conclusion

This study has shown that knowledge of maternal immunization among women (pregnant and non-pregnant), mothers and community leaders is fairly high and there is a general positive attitude towards maternal immunization in urban South Africa. To increase uptake of maternal immunization, antenatal and maternity staff, who are regarded as trusted source of information, need to be trained to provide adequate information regarding maternal immunization. Extending immunization information to everyone attending health facilities and not just pregnant women is crucially important as studies have shown that while men do not necessarily have an influence on the decision making regarding maternal immunization, pregnant women often seek advice from their mothers, peers or other family members (Willsam et al., 2019). Findings of this study established that when people have correct information and sufficient knowledge regarding maternal immunization, particularly from trusted sources, then this will allay most of the existing fears and misconceptions regarding immunization and could lead to increase in uptake of maternal immunization. Improved knowledge coupled with the already existing positive attitude towards maternal immunization may increase confidence in current maternal immunization programmes and the future ones.

### 5.1 Recommendations

Structured training on immunization should be provided for antenatal and maternity health care providers. Information on maternal immunization should be incorporated into antenatal classes and also put up in health facilities in the form of posters and information pamphlets for the attention of everyone visiting a health facility.

## Declarations

### Author contribution statement

N. Myburgh and S. Adedini: Conceived and designed the experiments; Contributed reagents, materials, analysis tools or data.

C. Cutland: Conceived and designed the experiments.

M. Godongwana: Performed the experiments; Analyzed and interpreted the data; Contributed reagents, materials, analysis tools or data; Wrote the paper.

N. Radebe: Performed the experiments.

### Funding statement

This work was supported by the Immunizing Pregnant Women and Infants Network (IMPRINT), United Kingdom.

### Data availability statement

Data will be made available on request.

### Declaration of interests statement

The authors declare no conflict of interest.

### Additional information

No additional information is available for this paper.

## Appendix A Supplementary data

SSI_Women in enrolled in maternal immunization trials SSI_Women in enrolled in maternal immunization trials

FGDs_Husbands and Mothers of Pregnant Women FGDs_Husbands and Mothers of Pregnant Women

KII_Antenatal & Maternity Staff KII_Antenatal & Maternity Staff

KII_Community leaders KII_Community leaders

KII_Pregnant Women KII_Pregnant Women
