# Supplementary material for: Knowledge and attitudes towards maternal immunization: perspectives from pregnant and non-pregnant mothers, their partners, mothers, healthcare providers, community and leaders in a selected urban setting in South Africa
Source: Heliyon. 2021 Jan 30;7(1):e05926. doi: 10.1016/j.heliyon.2021.e05926 (PMC7851332; doi:10.1016/j.heliyon.2021.e05926)
Supplement: KII_Antenatal & Maternity Staff [file mmc3.docx]

## Assessing community acceptancy and health facility preparedness for implementation of maternal immunisation programs in urban and rural South Africa

**Key Informant Interviews**

**Respondent groups:** Antenatal and maternity staff from community clinics and tertiary hospitals; other maternity health care providers, including doulas, community midwives, breast feeding consultants

Introduction

Good day, my name is…………. I am a…………….at the Respiratory and Meningeal Pathogens Research Unit (RMPRU), a Wits University research unit and a division of the Wits Health Consortium based at Chris Hani Baragwanath Hospital..

I would like you to participate in the study entitled: Assessing community acceptancy and health facility preparedness for implementation of maternal immunisation programs in urban and rural South Africa.

This study aims to explore community acceptancy and health facility preparedness for implementation of maternal immunisation programs in selected urban and rural settings in South Africa.

**Part 1: Community perceptions on maternal immunization**

**Maternal Immunisation**

1. What do you understand when you hear the term ‘Maternal Immunisation’?
2. What are the current immunizations given to pregnant women as part of routine care
   1. Probe: which vaccines do you think are given to pregnant women and why?
3. Who do you think maternal immunisation protects?
   1. Look for the following answers:
      1. Pregnant mother only
      2. Unborn baby only
      3. Newborn baby (up to ~3 months) only
4. Would you say that pregnant women are open to receiving vaccinations during their pregnancy?
   1. Yes/ No- Please explain why or why not
   2. Do you think pregnant women are more likely to accept a vaccine if it was available free of charge in clinic?
   3. Do you think pregnant women are more likely to accept a vaccine if it was available, but they had to pay for it, (<R150) in order to protect their baby?
5. Fears
6. Within the health system/community, do maternity staff/health providers have any fears or concerns regarding maternal immunization?
7. Please explain your answer
8. Views
   1. What are some of the common views that you hear in your community/health centre relating to maternal immunization?
   2. Please explain why you think they hold such views
9. Beliefs
   1. Are there any beliefs that are prevalent in your community relating to maternal immunization?
   2. Please elaborate
10. Misconceptions

What are the good and bad things that you hear relating to maternal immunization?

Please explain

**Part 2: Acceptability of maternal immunization**

1. Social factors
2. Do women accept maternal immunisation more easily than men?
   1. Please explain.
3. Do younger people accept maternal immunization more easily than older people?
   1. Please explain
4. Do people who live in urban areas accept maternal immunisation more easily than people who live in rural areas?
   1. Please explain
5. Cultural factors
6. What are some of the cultural beliefs that could facilitate acceptability of maternal immunization?

Please explain

1. What are some of the cultural beliefs that could impede acceptability of maternal immunization?

Please explain

1. Religious factors
2. What are some of the religious beliefs that could facilitate acceptability of maternal immunization?

Please explain

1. What are some of the religious beliefs that could impede acceptability of maternal immunization?

Please explain

1. Economic factors
   1. What are some of the economic factors that could acceptability of maternal immunization?

Please explain

- 1. What are some of the economic factors that could impede acceptability of maternal immunization?

Please explain
