# Supplementary material for: Knowledge and attitudes towards maternal immunization: perspectives from pregnant and non-pregnant mothers, their partners, mothers, healthcare providers, community and leaders in a selected urban setting in South Africa
Source: Heliyon. 2021 Jan 30;7(1):e05926. doi: 10.1016/j.heliyon.2021.e05926 (PMC7851332; doi:10.1016/j.heliyon.2021.e05926)
Supplement: KII_Community leaders [file mmc4.docx]

**Assessing community acceptancy and health facility preparedness for implementation of maternal immunisation programs in urban and rural South Africa**

# Semi-structured Interviews or Individual In-depth Interviews

# Respondent groups: Community leaders, including ward counsellors, church leaders

**Introduction**

Good day, my name is…………. I am a…………….at the Respiratory and Meningeal Pathogens Research Unit (RMPRU), a Wits University research unit and a division of the Wits Health Consortium based at Chris Hani Baragwanath Hospital. I would like you to participate in the study entailed: Maternal immunisation in South Africa: exploring acceptancy and preparedness for implementation of maternal immunisation programs in urban and rural sites in South Africa.

This study aims to explore community acceptancy and health facility preparedness for implementation of maternal immunisation programs in selected urban and rural settings in South Africa.

__________________________________________________________________________

**Socio-Demographic Information of respondent**

1.1. Gender:

__ Female

__ Male

__Other

1.2. Age (years):____

1.3. Race:

__ White

__ Indian

__ Black

__ Coloured

__Other (specify) ________________________

1.4. Language group:

__ Tswana

__ Zulu

__ Xhosa

__ Tsonga

__ Venda

__ Swazi

__ Ndebele

__ Sotho

__ Pedi

__ Other (Specify)_________________________

1.5. What is your preferred language?

__ English

__ Afrikaans

__ Other (specify)____________________

1.6. Highest level of education completed

__ Primary education

__ Some high school but didn’t complete

__ Further Education Training (FET)

__ Grade 12\Matric

__ Some high school but didn’t complete

__ Did not finish tertiary

__ Tertiary

__ College

__ Technical College

__ University

__Did not finish tertiary

__ Further Education Training (FET)

__ University Graduate

1.7. Current employment status

__ Employed full-time

__ Employed part-time

__ Unemployed

__ Volunteer work

__ Other (specify) _____________________________________

1.8. Occupation __________________________________________

1.9. Have you ever had children?

__Yes

__No

1.10. Do you have any children who are less than 5 years?

__ Yes

__ No

1.11. If yes, how many?____

**Part 1: Community perceptions on maternal immunization**

**Maternal Immunisation**

1. What do you understand when you hear the term ‘Maternal Immunisation’?
2. Please explain if you know of any immunisations that are currently given to pregnant women as part of routine care
   1. Probe: which vaccines do you think are given to pregnant women and why?
3. Who do you think maternal immunisation protects?
   1. Look for the following answers:
      1. Pregnant mother only
      2. Unborn baby only
      3. Newborn baby (up to ~3 months) only
4. Do you/Would you encourage pregnant women to receive a vaccine during pregnancy?
   1. Yes/ No- Please explain why or why not
   2. If a vaccine was available free of charge in clinic, would you encourage pregnant women to accept it?
   3. If vaccine was available, but you had to pay for it, (<R150) would encourage pregnant women t pay for it, in order to protect their baby?
5. Fears
6. In your community, do people have any fears or concerns regarding maternal immunization?
7. Please explain your answer
8. Views
   1. What are some of the common views that you hear in your community relating to maternal immunization?
   2. Please explain why you think they hold such views
9. Beliefs
   1. Are there any beliefs that are prevalent in your community relating to maternal immunization?
   2. Please elaborate
10. Misconceptions

What are the good and bad things that you hear relating to maternal immunization?

Please explain

**Part 2: Acceptability of maternal immunization**

1. Social factors
2. Do women accept maternal immunisation more easily than men?
   1. Please explain.
3. Do younger people accept maternal immunization more easily than older people?
   1. Please explain
4. Do people who live in urban areas accept maternal immunisation more easily than people who live in rural areas?
   1. Please explain
5. Cultural factors
6. What are some of the cultural beliefs that could facilitate acceptability of maternal immunization?

Please explain

1. What are some of the cultural beliefs that could impede acceptability of maternal immunization?

Please explain

1. Religious factors
2. What are some of the religious beliefs that could facilitate acceptability of maternal immunization?

Please explain

1. What are some of the religious beliefs that could impede acceptability of maternal immunization?

Please explain

1. Economic factors
   1. What are some of the economic factors that could facilitate acceptability of maternal immunization?

Please explain

- 1. What are some of the economic factors that could impede acceptability of maternal immunization?

Please explain
