# Supplementary material for: Knowledge and attitudes towards maternal immunization: perspectives from pregnant and non-pregnant mothers, their partners, mothers, healthcare providers, community and leaders in a selected urban setting in South Africa
Source: Heliyon. 2021 Jan 30;7(1):e05926. doi: 10.1016/j.heliyon.2021.e05926 (PMC7851332; doi:10.1016/j.heliyon.2021.e05926)
Supplement: KII_Pregnant Women [file mmc5.docx]

**Assessing community acceptancy and health facility preparedness for implementation of maternal immunisation programs in urban and rural South Africa**

**Focus Group Discussions Topic Guide**

**Respondent groups:** pregnant mothers

**Introduction**

Good day, my name is…………. And my colleagues are…………, and……… We are working at the Respiratory and Meningeal Pathogens Research Unit (RMPRU), a Wits University research unit and a division of the Wits Health Consortium based at Chris Hani Baragwanath Hospital.

We would like you to participate in the study entitled: Assessing community acceptancy and health facility preparedness for implementation of maternal immunisation programs in urban and rural South Africa.

This study aims to explore community acceptancy and health facility preparedness for implementation of maternal immunisation programs in selected urban and rural settings in South Africa.

**_____________________________________________________________________**

**Socio-Demographic Information of respondent (information to be collected for individual participant of the FGD)**

1.1. Age (years):____

1.2. Race:

__ White

__ Indian

__ Black

__ Coloured

__Other (specify) ________________________

1.3.Language group:

__ Tswana

__ Zulu

__ Xhosa

__ Tsonga

__ Venda

__ Swazi

__ Ndebele

__ Sotho

__ Pedi

__ Other (Specify)_________________________

1.4. What is your preferred language?

__ English

__ Afrikaans

__ Other (specify)____________________

1.5. Highest level of education completed

__ Primary education

__ Some high school but didn’t complete

__ Further Education Training (FET)

__ Grade 12\Matric

__ Some high school but didn’t complete

__ Did not finish tertiary

__ Tertiary

__ College

__ Technical College

__ University

__Did not finish tertiary

__ Further Education Training (FET)

__ University Graduate

1.6. Current employment status

__ Employed full-time

__ Employed part-time

__ Unemployed

__ Volunteer work

__ Other (specify) _____________________________________

1.7. Occupation __________________________________________

1.8. Have you ever had children?

__Yes

__No

1.9. Do you have any children who are less than 5 years?

__ Yes

__ No

1.10. If yes, how many?____

**Part 1: Perceptions on maternal immunization**

1. What do you understand when you hear the term ‘Maternal Immunisation’?
2. Please explain if you know of any immunisations that are currently given to pregnant women as part of routine care
   1. Probe: which vaccines do you think are given to pregnant women and why?
3. Did you receive any of these vaccinations?
4. Who do you think maternal immunisation protects?
   1. Look for the following answers:
      1. Pregnant mother only
      2. Unborn baby only
      3. Newborn baby (up to ~3 months) only
5. If you could receive a vaccine during your pregnancy, would you accept it?
   1. Yes/ No- Please explain why or why not
   2. If a vaccine was available free of charge in clinic, would you accept it?
   3. If vaccine was available, but you had to pay for it, (<R150) would you pay for it, in order to protect you/ your baby?

**Part 2: Community perceptions on maternal immunization**

- 1. **Fears**

Do you have any fears regarding maternal immunization?

Please explain what your fears are and why do you have them?

- 1. **Views**

What are some of the common views that you hear in your community relating to maternal immunization?

Please explain why you think they hold such views

- 1. **Myths**

Are there any myths that are prevalent in your community relating to maternal immunization?

Please elaborate

- 1. **Misconceptions**

What are the misconceptions that you hear relating to maternal immunization?

Please explain

**Part 2:**

**Acceptability of maternal immunization**

1. **Social factors**

1. Do women accept maternal immunisation more easily than men?
   1. Please explain.
2. Do younger people accept maternal immunization more easily than older people?
   1. Please explain
3. Do people who live in urban areas accept maternal immunisation more easily than people who live in rural areas?
   1. Please explain

2. **Cultural factors**

What are some of the cultural beliefs that could facilitate or impede acceptability of maternal immunization?

Please explain

3. **Religious factors**

What are some of the religious beliefs that could facilitate or impede acceptability of maternal immunization?

Please explain

4. **Economic factors**

What are some of the economic factors that could facilitate or impede acceptability of maternal immunization?

Please explain
